# Supplementary material for: Outcomes of isoniazid preventive therapy among people living with HIV in Kenya: A retrospective study of routine health care data
Source: PLoS One. 2020 Dec 2;15(12):e0234588. doi: 10.1371/journal.pone.0234588 (PMC7710039; doi:10.1371/journal.pone.0234588)
Supplement: S1 Fig — The peak in 2015–16 is attributed to both the development of IPT policy by the Ministry of health and 100-days rapid results initiative (RRI). (DOCX) [file pone.0234588.s001.docx]

S1 Fig. The use of IPT among PLHIV from 2011 to 2018 in Kenya- The peak in 2015-16 is attributed to both the development of IPT policy by the Ministry of health and 100-days rapid results initiative (RRI)
