# Supplementary figures and images for: Outcomes of isoniazid preventive therapy among people living with HIV in Kenya: A retrospective study of routine health care data
Source: PLoS One. 2020 Dec 2;15(12):e0234588. doi: 10.1371/journal.pone.0234588 (PMC7710039; doi:10.1371/journal.pone.0234588)

###
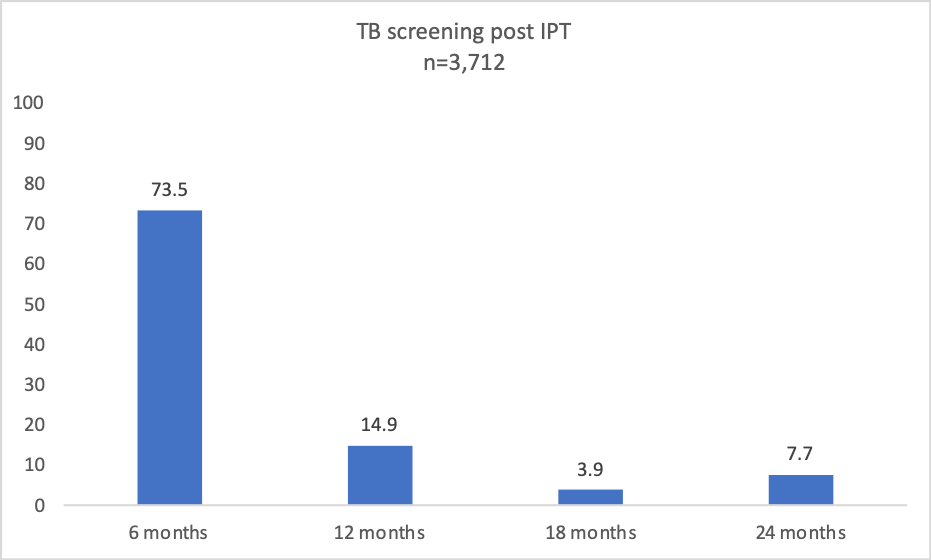


**S2 Fig.** Frequency of TB screening post IPT completion among PLHIV, Kenya, 2015-2018

Supplement: S2 Fig — (DOCX) [file pone.0234588.s002.docx]
