# Supplementary material for: Outcomes of isoniazid preventive therapy among people living with HIV in Kenya: A retrospective study of routine health care data
Source: PLoS One. 2020 Dec 2;15(12):e0234588. doi: 10.1371/journal.pone.0234588 (PMC7710039; doi:10.1371/journal.pone.0234588)
Supplement: Attachment — Submitted filename: Review_IPT outcomes_PLOS.docx [file pone.0234588.s004.docx]

**Outcomes of Isoniazid Preventive Therapy among people living with HIV in Kenya: A retrospective study of routine health care data**

**Summary**

**The authors conducted a retrospective review of records of people living with HIV (PLHIV) to determine the outcomes of isoniazid preventive (IPT) TB. The manuscript addresses a very important gap in the literature. Date are limited on the performance of the IPT cascade from high TB burden settings like Kenya. the authors report that two thirds of PLHIV on ART were initiated on IPT 95% of whom completed the six-month course.**

**Abstract**

**The abstract is well written and is a good summary of the manuscript. However, the first statement of the results which sounds as data collected outside of the described study. Because this statement is included in the main manuscript and contributes substantially to the conclusions I will elaborate on this comment in the results section of the manuscript.**

**Introduction**

**Paragraph 2 – the first statement refers to the TB burden in Kenya using TB incidence from WHO estimates. Since Kenya recently completed a TB prevalence survey and the results are available in published literature they should consider using this instead or in addition to the WHO estimates. Also when discussing the burden of TB/HIV co-infection in Kenya they refer to ministry of health reports. They should consider citing other analyses of Kenya TB surveillance data which have been published in peer reviewed journals as well.**

**Paragraph 3 – the authors have provided a list of specific objectives which could perhaps be summarized into fewer sentences.**

**Methods**

**Study setting: The authors should consider providing a more detailed summary of their study setting to enable readers to understand the study context. This could include information on how TB/HIV services are organized in Kenya especially IPT provision. What criteria was used to select the 30 counties that were included in this study?**

**Study population: "All PLHIV in care and initiated on IPT between 1st July 2015 through 30th June 2018"**

**This statement needs some clarification the authors included only clients on ART from specific facilities in specific counties. Also, before the implementation of test and treat approach to ART initiation, patients on care were not always on ART. Perhaps the word "care" should be replaced with "ART".**

**Data collection and management -**

**"The study team abstracted aggregate data for patients initiated**

**on IPT and PLHIV on ART" – this statement makes it sound as though the patients on IPT was a separate inclusion criteria from PLHIV on ART. Did the authors mean to say " PLHIV on IPT and ART"? Please revise this statement to reflect the study population.**

**Results**

1) **The currently described methods do not support this result: "We enrolled 138,442 PLHIV into ART during the study period and initiated 95,431 (68.9%) into IPT as per data retrieved from the National reporting warehouse." In fact, within this result the authors declare a different data source from what is described in the methods. If the authors wish to keep this result they need to revise the methods and include how they collected this data. Otherwise it is confusing how out of a sample size of roughly 3700 PLHIV a result of over 138,000 PLHIV is obtained. Also clarify whether these 138, 442 were from the entire country or from the study sites alone.**
2) **The presentation of results could be improved by having it structured into sections – description of study participants, IPT initiation and outcomes and factors associated with IPT completion**
3) **The authors should consider improving the logical flow of the results – its confusing to start with IPT outcomes, pedal back to screening which is a prerequisite to IPT initiation and back to outcomes (death and active TB)**
4) **Facility levels (2, 4, 5) are mentioned but have not been defined in the methods. Perhaps this also applies to facility ownership.**
5) **Figures and tables – the authors have included 3 figures and five tables. Perhaps some of these could be included as supplementary materials**

**Discussion and conclusions**

**Reference to the proportion of PLHIV on ART initiating IPT should be reviewed based on my earlier comment about this result being ectopic.**

**The authors should include their perspective on why IPT initiation was lower in higher level and private facilities.**

**References**

1. **The authors have relied a lot on non-peer reviewed sources (Ministry of Health and WHO). They should strive to rely more on peer reviewed sources if they are available.**
2. **The formatting of the references should be revised. Author names of institutions are abbreviated yet what these acronyms stand for is not spelt out (example – MOH).**
